# Quantitative proteomics analysis reveals an important role of the transcriptional regulator UidR in the bacterial biofilm formation of *Aeromonas hydrophila*

**DOI:** 10.3389/fcimb.2024.1380747

**Published:** 2024-03-22

**Authors:** Xiaoyan Li, Feng Tian, Binghui Zhang, Lishan Zhang, Xiaomeng Chen, Xiaoke Lin, Yuqian Wang, Xiangmin Lin, Yanling Liu

**Affiliations:** ^1^ College of Life Sciences, College of Juncao Science and Ecology, Fujian Agriculture and Forestry University, Fuzhou, China; ^2^ School of Life Sciences, Nanjing Agricultural University, Nanjing, China; ^3^ Key Laboratory of Marine Biotechnology of Fujian Province, Institute of Oceanology, Fujian Agriculture and Forestry University, Fuzhou, China; ^4^ National Engineering Research Center of Juncao Technology, Fujian Agriculture and Forestry University, Fuzhou, China; ^5^ Key Laboratory of Crop Ecology and Molecular Physiology, Fujian Agriculture and Forestry University, Fuzhou, China; ^6^ Institute of Tobacco Science, Fujian Provincial Tobacco Company, Fuzhou, China

**Keywords:** Aeromonas hydrophila, UidR, transcription regulator, biofilm formation, quantitative proteomics

## Abstract

**Introduction:**

Bacterial biofilm is a well-known characteristic that plays important roles in diverse physiological functions, whereas the current intrinsic regulatory mechanism of its formation is still largely unknown.

**Methods:**

In the present study, a label-free based quantitative proteomics technology was conducted to compare the differentially expressed proteins (DEPs) between *ΔuidR* and the wild-type strain in the biofilm state.

**Results:**

The results showed that the deletion of gene uidR encoding a TetR transcriptional regulator significantly increased the biofilm formation in *Aeromonas hydrophila*. And there was a total of 220 DEPs, including 120 up-regulated proteins and 100 down-regulated proteins between *ΔuidR* and the wild-type strain based on the quantitative proteomics. Bioinformatics analysis suggested that *uidR* may affect bacterial biofilm formation by regulating some related proteins in glyoxylic acid and dicarboxylic acid pathway. The expressions of selected proteins involved in this pathway were further confirmed by q-PCR assay, and the results was in accordance with the quantitative proteomics data. Moreover, the deletion of four genes (*AHA_3063, AHA_3062, AHA_4140* and *aceB*) related to the glyoxylic acid and dicarboxylic acid pathway lead to a significant decrease in the biofilm formation.

**Discussion:**

Thus, the results indicated that *uidR* involved in the regulatory of bacterial biofilm formation, and it may provide a potential target for the drug development and a new clue for the prevention of pathogenic *A. hydrophila* in the future.

## 1 Introduction

*Aeromonas hydrophila* is a conditionally pathogenic bacterium that distributed in a variety of aquatic systems, causing several serious diseases in fish, such as gastroenteritis, meningitis, endocarditis and bone marrow (Lin et al., 2017; Elbehiry et al., 2019; Akmal et al., 2020; Kumar et al., 2022). Currently, the abuse of antibiotics in aquaculture has led to the emergence of multi-drug resistant strains of *A. hydrophila*, which is becoming an increasingly serious problem and finally affects the human public health (Ahmed et al., 2018; De Silva et al., 2021; Zhang et al., 2022; Dorick et al., 2023) Therefore, it is essential to understand and explore the antibiotics resistance mechanism of *A. hydrophila* for its prevention and control. There are many reports on the bacterial antibiotic resistance mechanisms, such as biofilm formation, increasing efflux of antibiotics via efflux pumps, inactivation of antibiotic through modification, alteration of targeted sites of antibiotics, overproduction of exopolysaccharides, etc (Rasmussen-Ivey et al., 2016; Dias et al., 2017; Zhang et al., 2019; Seike et al., 2021).

Among these resistance mechanisms, biofilm formation has been reported to play an important role in bacterial antibiotic resistance, as it reduced the penetration of the antibiotics through a polysaccharide matrix and enhanced the physical defense system of cell membrane (Breser et al., 2018). In order to overcome antimicrobial hurdles, formation of various phenotypes by bacteria is a strategy for survival in complicated environments (Kussell and Leibler, 2005; Levin and Rozen, 2006). Biofilm, sessile communities of bacteria, has the high tolerance to stressors such as chemical sanitizers, salinity, and shear stress in aquatic environment (Dorick et al., 2023). Compared to planktonic state, bacteria in biofilm state could increase the capability of antibiotics resistance up to 1000 times (Hall et al., 2020). Moreover, bacterial biofilm also displays diverse resistance against environmental stresses and involves various important physiological activities of bacteria, such as global resistance, quorum sensing and virulence (Solano et al., 2014; Venkatesan et al., 2015; Dehbashi et al., 2023). Thus, a better understanding of how bacterial proteins regulate biofilm formation is necessary for the development of new antibiotic therapy strategies.

TetR family transcriptional regulators (TFRs) are the third largest family of transcriptional regulators in the bacterial genome to follow closely behind LTTRs and AraC, which are widely distributed in bacteria (Cuthbertson and Nodwell, 2013). They normally have an N-terminal DNA binding domain (about 50 amino acids) and a larger C-terminal ligand binding domain (Deng et al., 2013). For example, AcrR, a TetR-family transcriptional regulator which regulates the adjacent *acrAB* efflux genes, was reported to play an important role in ciprofloxacin resistance in *Escherichia coli* (Webber et al., 2018). Additionally, several TetR family transcription regulators, such as EmrR, EthR, QacR and RamR, have been found to be involved in the regulation of various important physiological functions in bacteria, including responding to osmotic stress, modification and removal of toxic compounds, regulation of catabolic pathways, influencing antibiotic production and virulence (Rkenes et al., 1996; Schujman et al., 2003; Kendall et al., 2010; Colclough et al., 2019). However, it is not understood that how TetR transcriptional regulators affect the regulation of bacterial biofilm formation.

Here, we reported the TetR family protein UidR (Uniport ID A0KQV4, gene ID *AHA_4233*), plays an important regulatory role in the biofilm formation in *A. hydrophila* ATCC 7966. This protein shares a moderate homology (40% identity) with UidR in *Escherichia coli* K12. The deletion of *uidR* significantly increased bacterial biofilm formation. To further understand the molecular regulatory mechanism of this protein, a label-free based quantitative proteomics technology was used to compare the differentially expressed proteins (DEPs) between the Δ*uidR* and its wild-type strain, and the following bioinformatics analysis showed that several metabolic pathways were altered in the mutant Δ*uidR* strain. Moreover, some selected related genes were deleted to assess their abilities of biofilm formation. In this study, we demonstrated the molecular regulatory mechanism of this TetR family protein on biofilm formation, and provided a new drug target candidate for the development of new therapy strategies against *A. hydrophila*.

## 2 Materials and methods

### 2.1 Strains, plasmids and growth conditions

In this study, the strains used were *A. hydrophila ATCC 7966* (wild type strain, WT), *E. coli* MC1061, *E. coli* S17 λpir, suicide vector pRE112 and shuttle vector pBBRMCS1, which were all kept in our laboratory (**Supplementary Table S1**). Both *E. coli* and *A. hydrophila* were cultured in fresh LB liquid medium, and incubated at 200 rpm 37°C and 30°C, respectively. When needed, chloramphenicol (Cm) and ampicillin (Amp) were added to the LB medium.

### 2.2 Construction of gene deletion mutant and complemented strains

The deletion of the target gene was performed using homologous recombination, as previously described (Wang et al., 2019). Briefly, about 500 bp upstream and 500 bp downstream sequences of the target gene was amplified and then fused in pRE112 suicide vector. The recombinant plasmid was transformed into *E. coli* MC1061 competent cells, and the plasmid of the positive clone was extracted and transformed into *E. coli* S17 competent cells. The *E. coli* S17 with recombinant plasmid was conjugated with WT at a ratio of 1:4 (v/v), and then screened in LB agar plate with 30 μg/mL Cm and 100 μg/mL Amp. The selected colonies were further screened and inoculated into the LB agar plate containing 20% sucrose or 30 μg/mL Cm, respectively. Finally, the positive colonies were picked up to verify by PCR and DNA sequencing, and then stored at −80 °C before use.

The complemented *ΔuidR* strain was constructed using ligase (Clon Express II One Step Cloning Kit, Vazyme) to ligate the gene sequence of with the digested pBBRMCS1 plasmid (*HindIII* and *BamHI*), producing a recombinant plasmid. Finally, the recombinant plasmid was transformed into *ΔuidR* strain by electroporation to generate the complemented strain of *ΔuidR*, which was finally confirmed by PCR and DNA sequencing. The pair primers for gene deletion and the construction of the complemented strain used in this study were listed in **Table 1**.

**Table 1:** Sequences of primer pairs used in this study for construction of the deletion strains and complemented strains.

Gene	Oligonucleotide sequence (5′ → 3′)	note
*AHA_4233*	P1-catgaattcccgggagagctcGGAGTTGATCTCCGGCATCC	upstream of target gene
	P2-gggcagtaAACTCCATGATCGTAGTAACTGGCG	
	P3-gatcatggagttTACTGCCCATTGTTGATAATCAGC	downstream of target gene
	P4-cgatcccaagcttcttctagaCCGTCGATGATGGAGGCG	
	P5-ccaccgagctgttcaacgatc	for gene validation
	P6-ccagttgacgatagtgctgttcc	
	P7-ccgtcgcagccttcgaacagc	for gene validation
	P8-cggtggcgatcagcttgaagc	
*AHA_3062*	P1-catgaattcccgggagagctcGGATCTGCTGGCCGACTACG	upstream of target gene
	P2-aggGAGATCAGGCTCCTACTTGGCG	
	P3-agtaggagcctgatctcCCTGCTGCTGGTGAGTACCG	downstream of target gene
	P4-cgatcccaagcttcttctagaCTTCATGAACCAGCGCACATC	
	P5-ttcatgtgtgacaccaagcgctg	for gene validation
	P6-aacagctcttcaccgctgcc	
	P7-ggcaagaactggaagaccgatc	for gene validation
	P8-ccgacgatgtggatgaagctgg	
*AHA_3063*	P1-catgaattcccgggagagctcTACTGATTGGCGAGCAGCACC	upstream of target gene
	P2-gcttggtgtGAGTTCTCCTTCACTGAGTCGGC	
	P3-aaggagaactcACACCAAGCGCTGCATCG	downstream of target gene
	P4-cgatcccaagcttcttctagaACCGTACTTCTGGCGCTCC	
	P5-atggtcaattccggtctggtgg	for gene validation
	P6-tgaccttggtctcctgcatctgg	
	P7-ttgatggagctgccgctggcag	for gene validation
	P8-ccgctcgcggaagatgtcgg	
*aceB(AHA_2816)*	P1-catgaattcccgggagagctcCCTACGTTATTGCGCCTCACTT	upstream of target gene
	P2-agccGGCAACACGATTCATAAGGTGC	
	P3-tatgaatcgtgttgccGGCTCGCCAATAACAAGTCAA	downstream of target gene
	P4-cgatcccaagcttcttctagaCCACTGGATCTGGTCGGCA	
	P5-ccactgcagcgctcgtctgc	for gene validation
	P6-tcagagccgctcgtaaccgg	
	P7-atgattactgagccgacggc	for gene validation
	P8-cgagctgatcttcgaagtgcacg	
*AHA_4140*	P1- catgaattcccgggagagctcGGAGTTGAGACGATCTTCGAGTTC	upstream of target gene
	P2-agacaactccCCGACTCTACGGTTGCCATT	
	P3-gtagagtcggGGAGTTGTCTGCACCTCATCAAC	downstream of target gene
	P4-cgatcccaagcttcttctagaAGTAGTGGCGCACCATCCG	
	P5-gcaatcgcatcacggaagtgg	for gene validation
	P6-cacgttcagcagcaggaactcc	
	P7-gcagatgcagcttgtcatgctcg	for gene validation
	P8-ggctggtcgatgtgcacgatac	
*AHA_4233* complemented	F-AGATCCTTGGAGTACTCGTAGTTGACCTCCATG	for gene validation
	R-TCACCCGGCTGGTTGGGCCAGTTGACGATAGT	

### 2.3 Bacteria growth measurement

The overnight bacteria were separately diluted 1:100 in fresh LB medium and thoroughly mixed, then 300 μl bacteria solution were added into the holes of the growth curve plate and cultured overnight for 16 h at 30°C. Bacterial growth was measured using a growth curve analysis system (Bioscreen C, BioRad) at 600nm. There were three independent replicates of this experiment.

### 2.4 Biofilm formation assay

The biofilm formation of bacterial strain was analyzed by crystal violet staining method, as previously described (Wang et al., 2019). In brief, the overnight bacterial strains were transferred to fresh LB medium at the ratio of 1:100 and incubated until the logarithmic growth phase (OD600nm ~1.0) at 200 rpm, 30 °C. The culture bacterial solution was then diluted at 1:100 and transferred to a 96-well microplate, and incubated at 30 °C for 24h stationary incubation. Then, the wells were washed slowly with double-distilled water for three times and then dried out in air. Subsequently, 250 μL of 0.1%crystal violet solution (w/v) was added in each well, and then stained at room temperature for 30 min. After that, the wells were rinsed slowly with double-distilled water for three times and then dried again. Finally, 300 μL of 95% ethanol solution (v/v) was added to each well to dissolve the dyed crystal violet, and the absorbance at 595 nm was detected using a SpectraMax^®^ i3 multifunctional microplate reader (Molecular Devices Corporation, California, USA). Each sample in the experiment was repeated in eight wells for technical replicates, and performed three independent times for biological replicates.

### 2.5 Biofilm protein extraction and digestion

The preparation of protein in biofilm was performed as previously described (Li et al., 2016). Briefly, the overnight bacterial strain was transferred to fresh 5 ml LB medium at the ratio of 1:100, and then incubated at 30°C to an OD_600_ of 1.0. The cultured bacterial solution was diluted at 1:200 in a polystyrene Petri dish (Fisher Scientific, Franklin, MA) and incubated at 30°C for 24h stationary incubation. After being washed carefully three times with PBS, the mature biofilm protein was obtained by leaching twice with PBS buffer. The bacterial cells were then washed three times with PBS and centrifuged at 12,000 rpm, 30 min, 4°C, and resuspended in lysis buffer (6 M urea, 2 M thiourea, 0.1 M Tris-HCl (pH 8.5), protease inhibitor). The bacterial suspensions were then ruptured by sonication at 4°C for 15 mins and centrifuged (12,000 rpm, 30min, 4°C) to collect the supernatant. The supernatant was transferred to a new tube, and the protein concentration was determined by Bradford assay. Approximately, 50 μg extracted protein from each group was reduced with 100 mM dithiothreitol (DTT) for 1 hour at room temperature, and alkylated with 50 mM iodacetamide (IAA) in the dark for 30 min in UT buffer (8 M urea, 0.1 M Tris, pH 8.5). The proteins were then digested by trypsin (Promega Corporation, Madison, USA) at 1:50 ratio by a FASP (Filter aided sample preparation) method, as previously descried (Wisniewski, 2016). The digested peptides were desalted by a C18 column (Waters, Massachusetts, USA) and then dried down by a CentriVap concentrator (Labconco Corporation, Missouri, USA).

### 2.6 Quantitative analysis by LC−MS/MS

The digested peptides were dissolved in 0.1% Formic acid (FA) and separated on an EASY-nLCTM 1200 UHPLC system (Thermo Fisher Scientific, Massachusetts, USA) and analyzed with an Orbitrap Q Exactive HF-X mass spectrometer (Thermo Fisher Scientific, Massachusetts, USA) as previously described (Zhang et al., 2020). The scanning mode of mass spectrometry is data-dependent acquisition (DDA), with an electrospray voltage of 2.3 kV, a capillary temperature was 320 °C, and a full MS scan with a scan range of 350 to 1500 m/z and automatic gain control (AGC), a maximum ion implantation time of 45 ms, a normalized collision energy of 27%, an intensity threshold of 8.3×10^3^, and a dynamic exclusion parameter set to 60 s. Each sample in this experiment was repeated in three for technical replicates, and performed three independent times for biological replicates.

The raw MS data were searched using Maxquant 1.6.17.0 against *A. hydrophila* ATCC 7966 Uniport database. Proteins with a protein abundance ratio difference greater than 1.5 or less than 0.667 and a *P value <*0.05 were considered as differentially expressed proteins (DEPs) and submitted for subsequent bioinformatics analysis.

### 2.7 Bioinformatics analysis

Gene Ontology (GO) annotations were classified and enriched according to GO terms of biological processes (BP), cell components (CC), and molecular functions (MF). Briefly, DAVID online software (https://david.ncifcrf.gov/) was used to perform GO enrichment analysis of DEPs, and then the GO plot package in the R language software was performed to visualize (Damian et al., 2015; Li et al., 2020). The KEGG pathways in differential expressed proteins were enriched and analyzed by the online tool OmicsBean (http://www.omicsbean.cn/) and then visualized with the GO plot package in the R language software. The prediction of protein-protein interaction (PPI) of DEPs network was analyzed by STRING version 11.5 (https://string-db.org/) and visualized using Cytoscape version 3.8.2 (Shannon et al., 2003; Damian et al., 2018).

### 2.8 qPCR assay

The qPCR assay of biofilm was performed as previously described (Cai et al., 2019). Briefly, the total RNA of each bacterial sample in biofilm state was extracted according to the TRIzol-chloroform (Takara standard Co. LTD., Japan) extraction method. Then, the total RNA was reverse-transcribed into cDNA according to the operating steps of the Prime Script RT reagent Kit (TransGen Biotech, Beijing, China)instructions. Finally, the real-time fluorescent quantitative PCR was used to detect the transcription regulation-related genes using the Real-Time PCR Detection System (Bio-Rad, California, USA) instrument with the pair primers listed in **Supplementary Table S2**. Each sample in this experiment was repeated in eight wells for technical replicates, and performed three independent times for biological replicates.

## 3 Results

### 3.1 The deletion of *uidR* affects the biofilm formation in *A. hydrophila*

**Figure 1:**
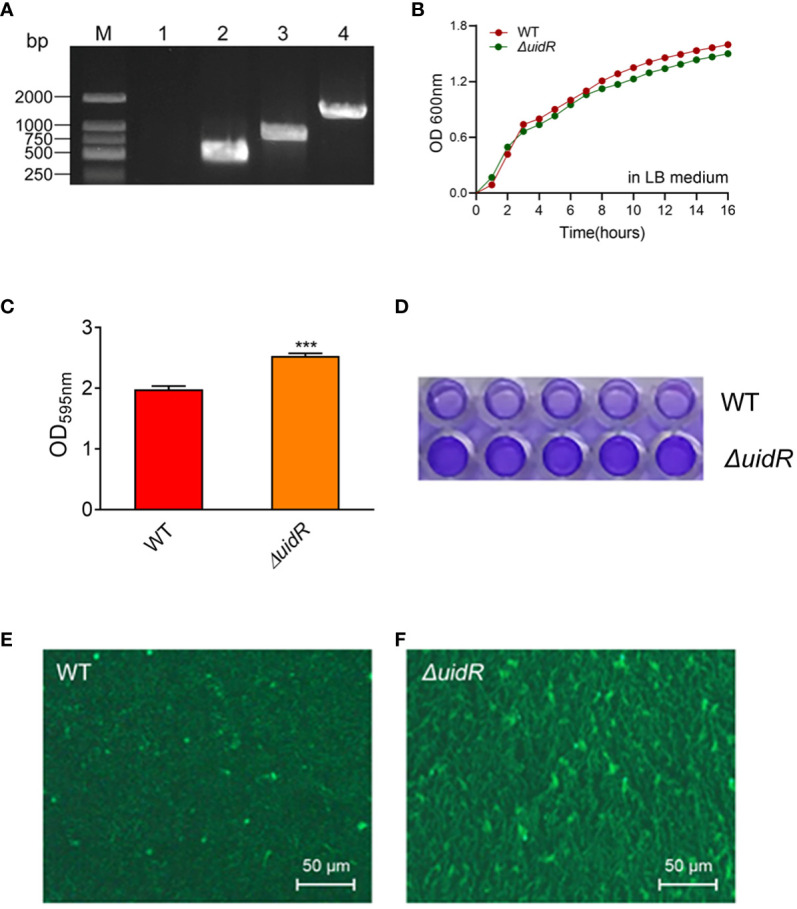
The validation of *uidR* knockout mutant and the biofilm formation ability in WT and ΔuidR strains. **(A)** M: DL2000 maker; Lane 1 to 2, the fragments of target gene DNA amplified using the primer pair P5/P6 in *ΔuidR* and WT strain, respectively; Lane 3 to 4, the fragments of target gene DNA amplified using the primer pair P7/P8 in Δ*uidR* and WT strain, respectively. **(B)** Growth curves of Δ*uidR* and WT strains in LB medium. **(C)** Quantification of biofilm formation (OD595nm) by CV staining, ***P<0.001. **(D)** Determination of biofilm biomass on polystyrene surface stained by CV. **(E, F)** Observation of the surface morphology of biofilms of *WT* and Δ*uidR* by the Zeiss vertical fluorescence microscope, respectively.

The *ΔuidR* strain was successfully constructed using a two-step homologous recombination method. As shown in **Figure 1A**, when compared to the wild-type (WT) strain, the DNA fragment of *uidR* was absent in *ΔuidR* strain using the PCR pair primers (P5P6) for target gene amplification. Additionally, the band of PCR product of *ΔuidR* strain was significantly lower than WT strain when using PCR pair primers (P7P8) to amplify the upstream and downstream range of the target gene, indicating that the knocked-out strains were successfully constructed. The positive clone was selected and further confirmed by DNA sequencing.

In order to investigate the effect of the *uidR* deletion in *A. hydrophila*, the bacteria growth and the biofilm formation were measured. As shown in **Figure 1B**, the growth between the *ΔuidR* and WT strain had no significant difference. Meanwhile, the crystal violet staining analysis showed that the *ΔuidR* strain significantly increased the ability of biofilm formation when compared to WT strain (*P<0.001*) (**Figures 1C, D**). This was further confirmed by fluorescence microscope using acridine orange staining method, which showed that the fluorescent intensity of *ΔuidR* in biofilm was significantly stronger than WT (**Figures 1E, F**).

**Figure 2:**
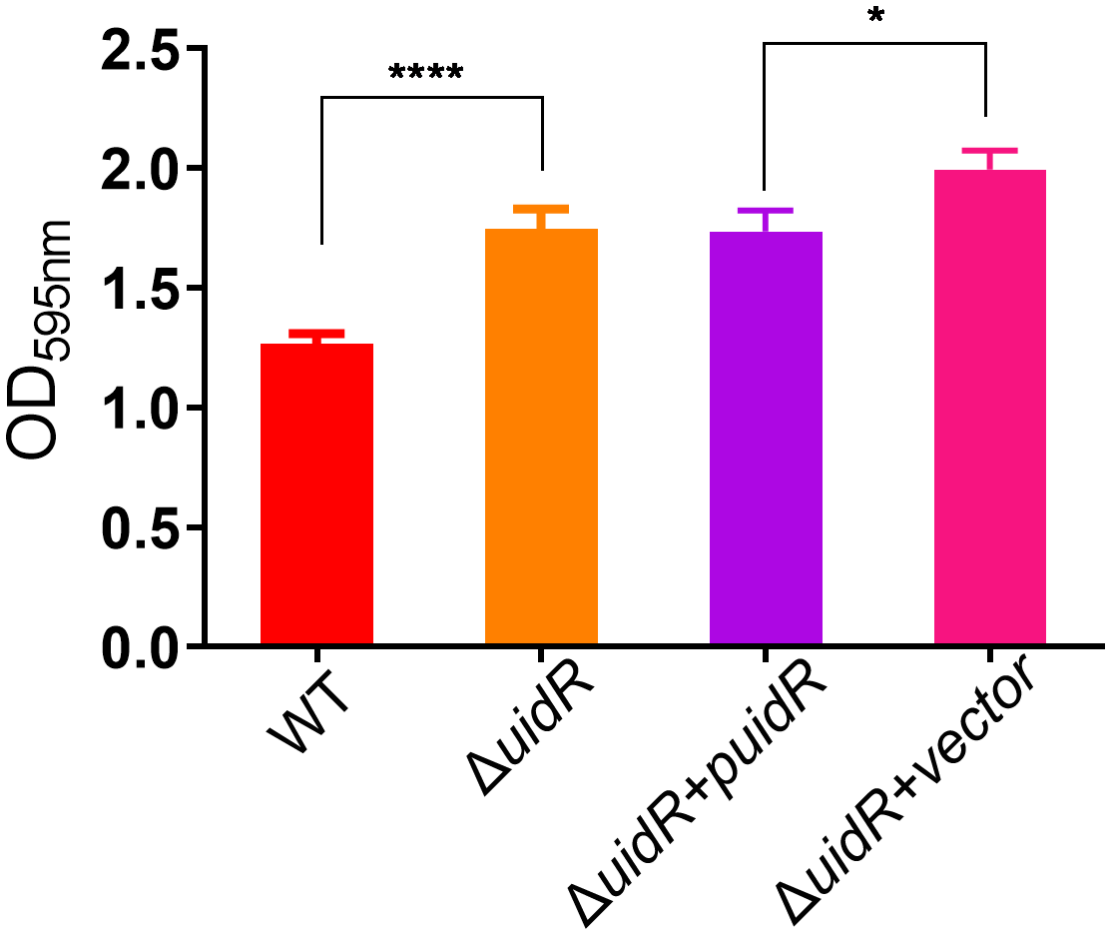
The biofilm formation ability of *ΔuidR* and its complemented strain, with *WT* and *ΔuidR* carrying empty vector as negative controls. Error bars represent the standard error of the mean (SD). Asterisks indicate significant differences in T-test between WT and Δ*uidR*, complemented strain (Δ*uidR* +p*uidR*) and Δ*uidR* carrying empty vector (Δ*uidR* +vector), *P <0.05, **** *P* <0.0001.

Moreover, the complemented strain was validated by PCR (**Supplementary Figure S1**) and DNA sequencing, and the property of biofilm formation in the complemented strain was restored to the WT level (**Figure 2**). These data indicated that the deletion of *uidR* negatively affected the *A. hydrophila* biofilm formation, and inferred that the TetR family transcriptional regulator may play an important role in the regulation of the biofilm formation in *A. hydrophila*.

### 3.2 Proteomics analysis and comparison of *WT* and *ΔuidR* in biofilm

**Figure 3:**
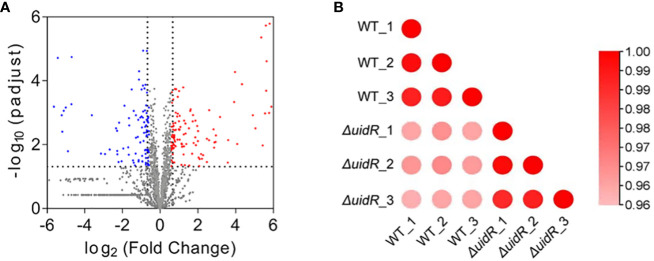
Label-free quantitative proteomics data analysis between *ΔuidR* and WT strain in biofilm. **(A)** Volcano plots comparing the abundance ratios of proteins between *ΔuidR* and WT strain in biofilm. The gray dots represent non-differentially abundant proteins, the blue dots represent differentially down-regulated proteins and red dots represent differentially up-regulated proteins; **(B)** Correlation coefficient to analyze the correlation of protein intensity in three biological replicates of each group.

To further investigate the role of *uidR* as a transcriptional regulator, we used a label-free quantitative proteomics method to compare the DEPs of the *ΔuidR* and WT strain. As shown in **Figure 3A**, a total of 1496 proteins were identified by mass spectrometry, with 220 proteins being differentially expressed between the *ΔuidR* and WT strain, including 120 up-regulated and 100 down-regulated proteins in the *ΔuidR* strain. Moreover, the correlation analysis of protein intensity among three biological repeats in each group showed that these samples had a high reproducibility with a correlation coefficient >0.96, indicating the stability and reliability of the proteomics data (**Figure 3B**).

### 3.3 GO and KEGG enrichment analysis of differentially expressed proteins

**Figure 4:**
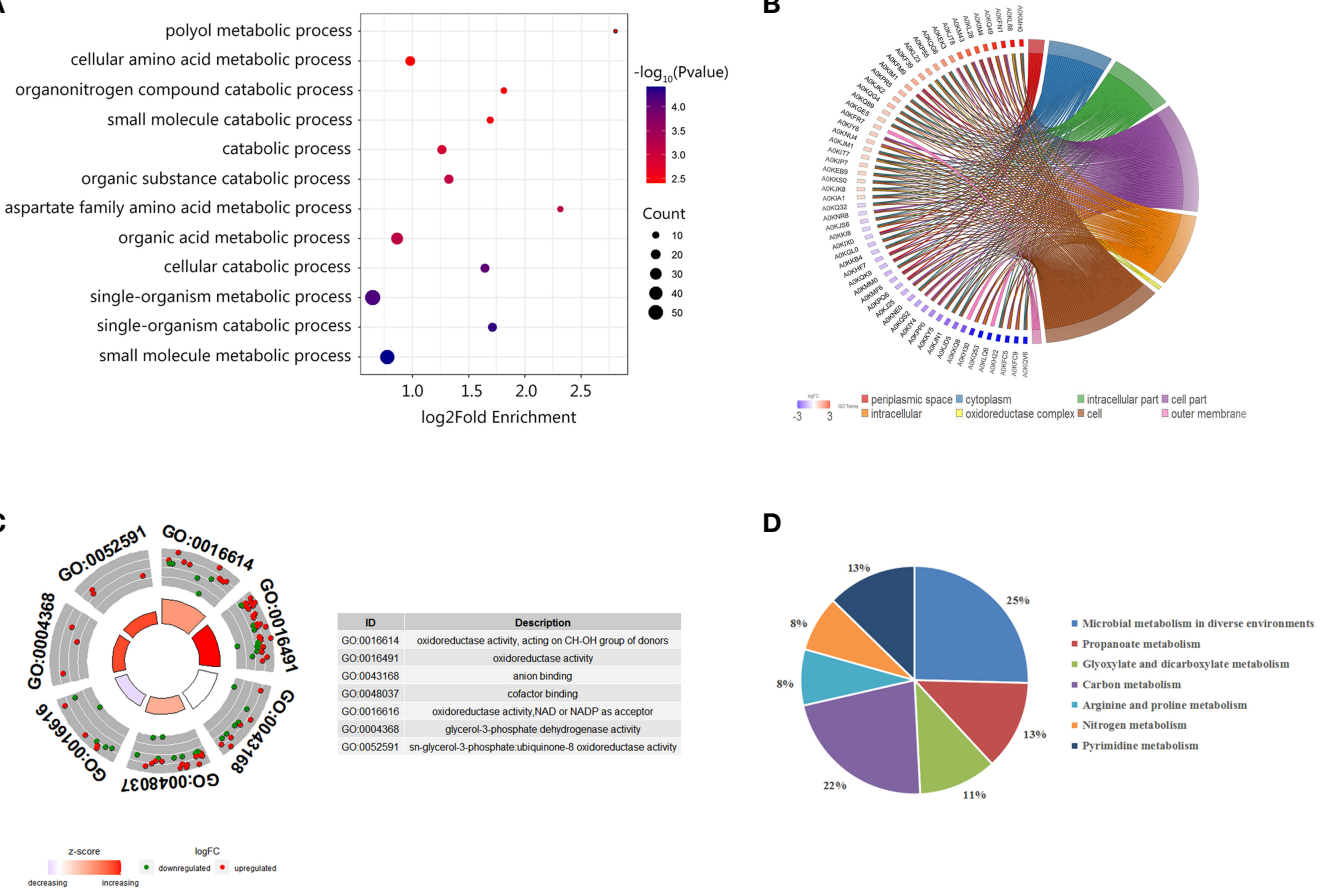
Enrichment analysis of differentially abundant proteins between Δ*uidR* and WT strain in biofilm status. **(A)** GO enrichment analysis in biological process; **(B)** Enrichment analysis in the cell component; **(C)** GO enrichment analysis molecular function; **(D)** KEGG enrichment analysis of differentially abundant proteins.

To further understand the functions of these identified proteins, we classified the 220 DEPs by Gene Ontology (GO) categories, including biological process (BP), cell component (CC), and molecular functioning (MF). As showed in **Figure 4A**, the DEPs participated in diverse biological functions in the BP classification. Among DEPs, 55 proteins were involved in a single-organism metabolic or catabolic process, followed by 47 DEPs that were involved in small molecule metabolic process. According to the CCs enrichment, most of the DEPs were distributed in the cytoplasm, some were distributed in the oxidoreductase complex and the outer membrane (**Figure 4B**). As shown in the MF classification (**Figure 4C**), the increasing abundance proteins were mostly enriched in the oxidoreductase activity, cofactor binding, CH-OH group acting on the donor in the activity, and the glycerol-3-phosphate dehydrogenase activity and ubiquinone-8 oxidoreductase activity. The decreasing abundance proteins were preferring to NAD or NADP as the acceptor, and CH-OH group as the donor processes, while anion binding process presented unbiased.

We further performed KEGG enrichment analysis on DEPs, and a total of 63 proteins were enriched, which participated in a total seven metabolic pathways. As shown in **Figure 4D**, 25% of DEPs was involved in the metabolic pathways of microorganisms in diverse environments, 22% for the carbon metabolite pathway, 13% for the propionate metabolic pathway, 13% for the pyrimidine metabolic pathway, and 11% for glyoxylate and dicarboxylate metabolism. Since previous research had reported that the proteins related to glyoxylate and dicarboxylate metabolism pathway were involved in bacterial biofilm formation in *Marinobacter hydrocarbonoclasticus* (Vaysse et al., 2009), we were then interested in the role of these proteins on *uidR* mediated biofilm formation in this study.

### 3.4 The glyoxylic acid and dicarboxylic acid pathway affect the biofilm formation of *A. hydrophila*

**Figure 5:**
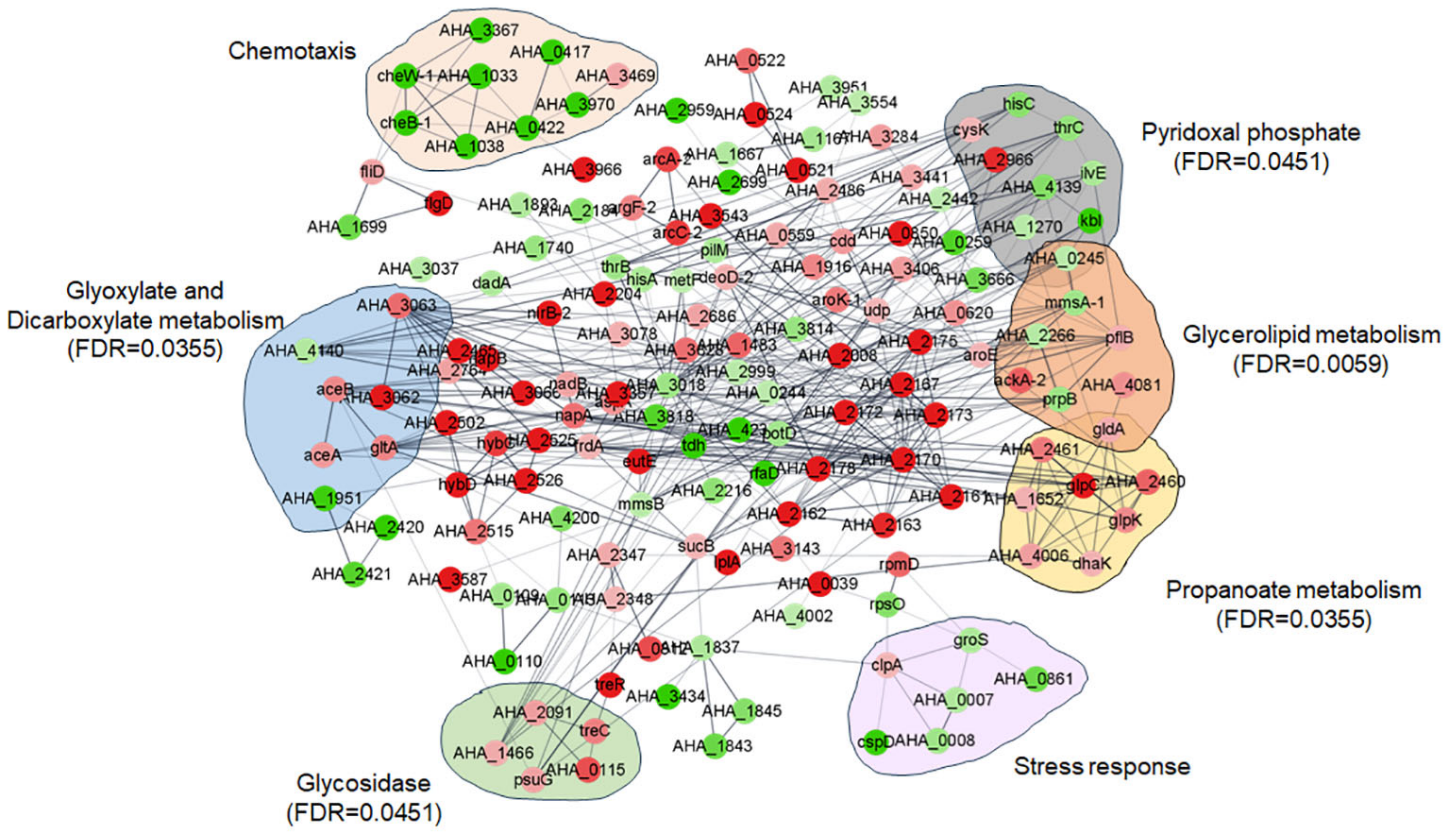
Protein and protein interaction network (PPI) of between WT and *ΔuidR* in biofilm status.

PPI relationships among the DEPs between the *ΔuidR* and WT strain in biofilm were established based on the STRING online software. As shown in **Figure 5**, nine DEPs (*cysk*, *hisC*, *thrC*, *AHA_2966*, *AHA_4139, ilvE, AHA_1270, kbl, AHA_0245*) were mainly enriched in pyridoxal phosphate (FDR=0.0451), eight DEPs (*AHA_0245*, *mmsA-1*, *AHA_2266*, *pflB*, *ackA-2*, prpB, *AHA_4081*, *gldA*) were mostly enriched in glycerolipid metabolism (FDR=0.0059), five DEPs (*AHA_2091*, *treC*, *AHA_1466*, *psuG*, *AHA_0115*) were enriched in glycosidase (FDR=0.0451) and eight DEPs (*gldA*, *AHA_2461*, *AHA_2460*, *glpC*, *AHA_1652*, *glpK, AHA_4006*, *dhaK*) were enriched in propanoate metabolism (FDR=0.0355). Interestingly, there were still a series of proteins with similar functions showing significant differences in some pathways which had no different significance in the FDR value, such as nine DEPs (*AHA_3367*, *AHA_0417*, *cheW-1*, *cheB-1*, *AHA_1033*, *AHA_3970*, *AHA_0422*, *AHA_1038*, *AHA_3469*) in chemotaxis and six DEPs (*clpA*, *groS*, *AHA_0007*, *AHA_0861*, *cspD*, *AHA_0008*) in stress response.

**Figure 6:**
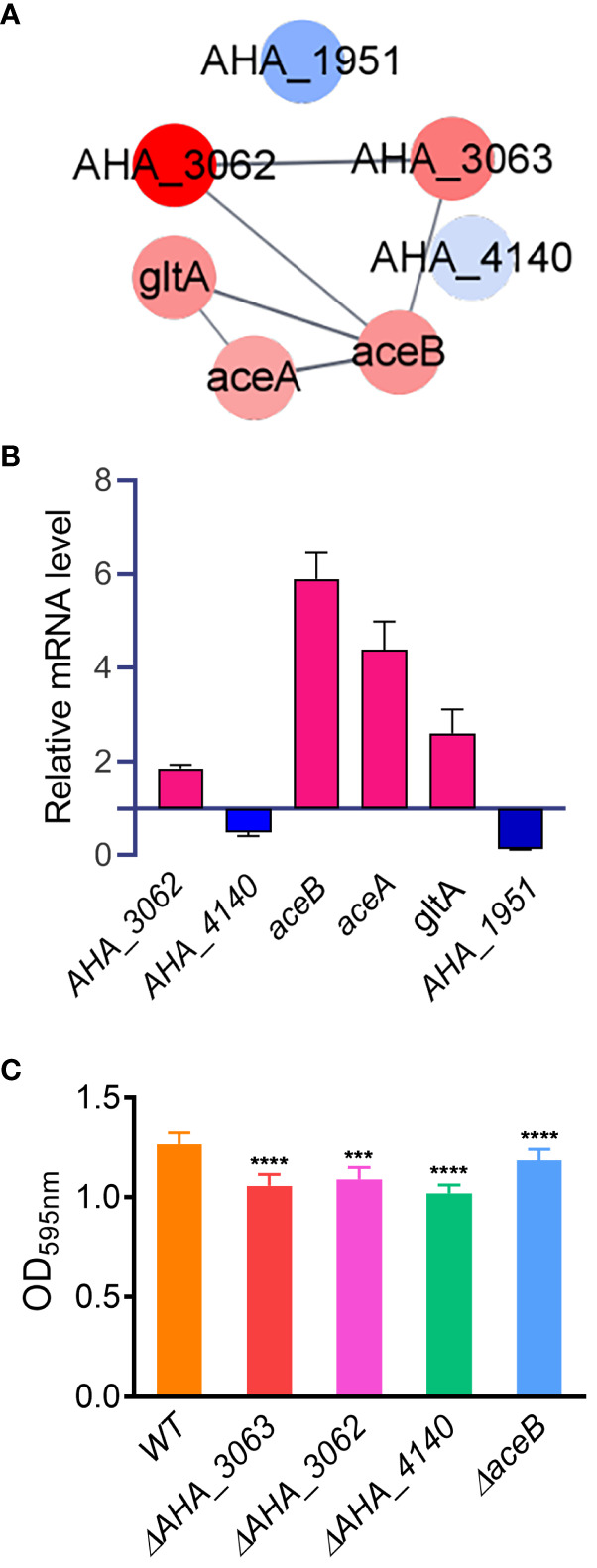
The glyoxylic acid and dicarboxylic acid pathway affects the biofilm formation of *A hydrophila*. **(A)** Protein-protein interaction prediction of altered proteins in glyoxylate and dicarboxylate metabolism pathway. Red color indicates up-regulation of protein; blue color indicates down-regulation of protein; **(B)** The mRNA levels of six glyoxylate and dicarboxylate metabolism pathway related genes were analyzed by qPCR method between *ΔuidR* and *WT* strain in biofilm; **(C)** The biofilm formation ability of several glyoxylic acid and dicarboxylic acid metabolism related mutants. Error bars represent the standard error of the mean (SEM). Asterisks indicate differences in T-test between WT and mutant strains. *** *P* <0.001; **** *P* <0.0001.

Furthermore, PPI analysis also showed that seven altered proteins including two down-regulated proteins (*AHA_1951 and AHA_4140*), and five up-regulated proteins (*AHA_3062*, *AHA_3063*, *aceA*, *aceB* and *gltA*) were involved in glyoxylate and dicarboxylate metabolism pathway (FDR=0.0355) (**Figure 6A**). The results showed that most of DEPs in this pathway interacted with each other, and most of them were up-regulated in *uidR* deletion strain, indicating *uidR* may negatively regulate the expression of these proteins during biofilm formation in *A. hydrophila.*

To further validate the accuracy of the proteomics data, six genes involved in the glyoxylate and dicarboxylate metabolism pathway (*AHA_1951*, *AHA_4140*, *AHA_3062*, *aceA*, *aceB* and *gltA*) were selected to compare their mRNA levels between the Δ*uidR* and *WT* strain in biofilm by qPCR method. As shown in **Figure 6B**, when compared to *WT* strain, the transcription of *AHA_3062*, *aceA*, *aceB* and *gltA* were significantly increased, while *AHA_4140* and *AHA_1951* were significantly decreased in biofilm formation, being consistent with the proteomics results. And the results indicated the reliability of the quantitative proteomics data.

In order to further verify the effects of proteins related to the glyoxylic acid and dicarboxylic acid metabolic pathway on the biofilm formation of *A. hydrophila*, four genes (*AHA_3062*, *AHA_3063*, *AHA_4140* and *aceB*) involved in this pathway were deleted to evaluate their biofilm formation capabilities by crystal violet staining method. As shown in **Figure 6C**, the deletion of these four genes significantly reduced the biofilm formation capability in *A. hydrophila* when compared with *WT* stain, respectively. These results further validated the role of the genes related to toglyoxylate and dicarboxylate metabolism in biofilm formation. Moreover, since the proteins encoded by these genes were up-regulated in *ΔuidR* strain in proteomics results, suggesting that the TetR family transcription factor may negatively regulate glyoxylate and dicarboxylate metabolism related genes and then affect the biofilm formation in *A. hydrophila*.

## 4 Discussion

Biofilm formation is a complexed physiological process in which bacterial aggregates are formed and stabilized by extracellular polymer substrates (EPS). The structure provides protection for microorganisms from external harsh environments, such as nutrients starvation, changes in pH, osmotic pressure, and antibiotics stress (Kim et al., 2012). The expression of genes or proteins related to other cellular activities, such as metabolism, transport, virulence, quorum sensing, exercise, and stress response, has been found to be involved in the regulation of biofilm formation (Park et al., 2014; Basic et al., 2017). The family of TetR transcriptional regulators is the third largest family of prokaryotic regulators, and previous research had documented their importance in the diverse physiological functions associated with biofilm formation (Bhukya, 2017). However, the underlying molecular mechanism by which TFRs trigger the expression of related genes or proteins during biofilm formation remains elusive.

In this study, we firstly identified the transcriptional regulator UidR in *A. hydrophila* as a factor that significantly improved the ability to form biofilms while there was no significant difference between *ΔuidR* and WT strain in the bacteria growth. To better understand the intrinsic molecular regulatory mechanism of this transcriptional factor on biofilm formation, a label-free based quantitative proteomics method was performed to compare the DEPs between *ΔuidR* and WT strain in *A. hydrophila* biofilm, including 120 up-regulated and 100 down-regulated proteins.

Bioinformatics analysis showed that UidR regulated the expression of proteins related to several metabolic pathways that were involved in the biofilm formation. Most of these DEPs were enriched in the carbon metabolism pathways, including propanoate metabolism, glyoxylate and dicarboxylate metabolism, and most proteins related to metabolic pathways, especially the carbon metabolism pathway, and up-regulated in the *ΔuidR* biofilm. Based on the fact that carbon availability plays an important role in the biofilm architecture (Bester et al., 2011), it was reasonable that the proteins related to the carbon metabolic were up-regulated in this study. Interestingly, several studies have suggested that the glyoxylate and dicarboxylate metabolic pathway may be involved in the bacterial biofilm formation. For example, exogenous glyoxylic acid supplementation had been shown to increase biofilm formation in *Pseudomonas aeruginosa* (Bahamondez-Canas and Smyth, 2018). Meanwhile, the significant changes of glyoxylate and dicarboxylate metabolism during biofilm formation had been reported in various bacterial species, such as *P. aeruginosa*, avian pathogenic *E. coli* (APEC) and Methicillin-resistant *Staphylococcus aureus* (MRSA) using omics technologies (D’Arpa et al., 2021; Ruan et al., 2021; Zhang et al., 2021). Moreover, the mutations of key enzymes in the glyoxylate cycle have been found to affect bacterial biofilm formation, and the deletion of isocitrate lyase gene *icl1* in *Candida albicans* reduced biofilm formation on acetate, lactate, ethanol and oleic acid as carbon sources (Shu et al., 2019). Furthermore, the biofilm formation of citrate synthase *gltA* mutants, which are also tricarboxylic acid (TCA) cycle key enzymes, was significantly decreased in *Staphylococcus aureus* USA300 (Backer et al., 2018). Besides, several glyoxylate cycle-related proteins, such as formate dehydrogenase, malate synthase in other species even in fungi, have been reported to affect biofilm formation directly or indirectly (Pires et al., 2016; Pometun et al., 2020). Therefore, we are interested in the role of the proteins related to the glyoxylate and dicarboxylate metabolic pathway on biofilm formation.

In the present study, seven proteins involved in glyoxylate and dicarboxylate metabolism pathway were differentially expressed in *ΔuidR* strain when compared to WT. Glutamine synthetase A0KQL2 (*AHA_4140*) and acetoacetyl-CoA reductase A0KJN1 (*AHA_1951*) were down-regulated, while formate dehydrogenase A0KMR3 (*AHA_3062*) and A0KMR4 (*AHA_3063*), malate synthase AceB (*aceB*), isocitrate lyase AceA and citrate synthase GltA were up-regulated. The qPCR assay results suggested that *uidR* may regulate the glyoxylate and dicarboxylate pathway in biofilm formation in *A. hydrophila.* To verify our hypothesis, three up-regulated genes (*AHA_3063*, *AHA_3062* and *aceB*) were knocked out respectively, and their biofilm forming abilities were evaluated. The results showed that these mutants did significantly reduce the biofilm formation. Therefore, we concluded that the glyoxylate cycle has an important role in biofilm formation, and UidR may negatively regulate glyoxylate cycle pathway in biofilm formation of *A. hydrophila*.

In addition, it is particularly noteworthy that several DEPs have been reported to participate in the biofilm formation in other bacterial species. For example, Xanthine dehydrogenase and hypoxanthine oxidase are well known to participate in purine salvage pathway to product (p) ppGpp, which has been reported to be a guanosine nucleotide-based second messenger involved in bacterial biofilm formation (Paz et al., 2012; Gallegos-Monterrosa et al., 2017; Smitha et al., 2021). Ornithine carbamoyltransferase (OTC) is involved in the arginine deiminase system and an *otc*-deficient mutant in *Streptococcus suis* was found to reduce the extracellular matrix of biofilm as well (Li et al., 2020). In the present study, two xanthine dehydrogenases proteins (A0KK90 and A0KKA1), possible hypoxanthine oxidase XdhD, and two ornithine carbamoyl transferases proteins (A0KK84 and ArgF) were significantly upregulated in *uidR* mutant strain, indicating the important role of these proteins in biofilm formation in *A. hydrophila*. However, the intrinsic regulatory mechanism regulated by this TetR family regulator and biological function in biofilm formation need to be further investigated, it may provide a potential target for the drug development and a new clue for the prevention of pathogenic *A. hydrophila* in the future.

## Data availability statement

The datasets presented in this study can be found in online repositories. The names of the repository/repositories and accession number(s) can be found below: http://www.proteomexchange.org/, PXD034710.

## Author contributions

XYL: Writing – original draft, Formal analysis, Methodology. FT: Methodology, Writing – original draft, Validation, Writing – review & editing. BZ: Writing – original draft, Software, Visualization. LZ: Conceptualization, Methodology, Supervision, Writing – review & editing. XC: Data curation, Methodology, Visualization, Writing – original draft. XKL: Investigation, Software, Writing – original draft. YW: Formal analysis, Methodology, Software, Visualization, Writing – original draft. XML: Conceptualization, Funding acquisition, Project administration, Writing – original draft, Writing – review & editing. YL: Conceptualization, Funding acquisition, Project administration, Validation, Writing – original draft, Writing – review & editing.

## Supplementary material

Supplementary Table 1 The strains and plasmids used in this study.

Supplementary Table 2 Primers designed in this study for qPCR.

## References

[B1] Ahmed H. A. Mohamed M. E. M. Rezk M. M. Gharieb R. M. A. Abdel-Maksoud S. A. (2018). *Aeromonas hydrophila* in fish and humans; prevalence, virulotyping and antimicrobial resistance. Slovenian Veterinary Res. 55, 113–112. doi: 10.26873/SVR-636-2018

[B2] Akmal M. Rahimi-Midani A. Hafeez-Ur-Rehman M. Hussain A. Choi T. J. (2020). Isolation, characterization, and application of a bacteriophage infecting the fish pathogen *Aeromonas hydrophila* . Pathogens (Basel, Switzerland) 9, 215. doi: 3390/pathogens9030215 32183136 10.3390/pathogens9030215 PMC7157608

[B3] Backer S. D. Sabirova J. Pauw I. D. Greve H. D. Hernalsteens J. P. Goossens H.. (2018). Enzymes catalyzing the TCA-and urea cycle influence the matrix composition of biofilms formed by methicillin-resistant *Staphylococcus aureus* USA300. Microorganisms 6, 113. doi: 10.3390/microorganisms6040113 30380651 PMC6313315

[B4] Bahamondez-Canas T. Smyth H. D. C. (2018). Influence of excipients on the antimicrobial activity of tobramycin against *Pseudomonas aeruginosa* biofilms. Pharm. Res. 35, 10. doi: 10.1007/s11095-017-2301-5 29294187

[B5] Basic A. Blomqvist M. Dahlén. G. Svensäter G. (2017). The proteins of Fusobacterium spp. involved in hydrogen sulfide production from L-cysteine. BMC Microbiol. 17, 16. doi: 10.1186/s12866-017-0967-9 28288582 PMC5348791

[B6] Bester E. Kroukamp O. Hausner M. Edwards E. A. Wolfaardt G. M. (2011). Biofilm form and function: carbon availability affects biofilm architecture, metabolic activity and planktonic cell yield. J. Appl. Microbiol. 110, 387–398. doi: 10.1111/jam.2011.110.issue-2 21122038

[B7] Bhukya H. (2017). TetR regulators: a structural and functional perspective. J. Indian Institute Sci. 97, 1–15. doi: 10.1007/s41745-017-0025-5

[B8] Breser M. L. Felipe V. Bohl L. P. Orellano M. S. Isaac P. Conesa A.. (2018). Chitosan and cloxacillin combination improve antibiotic efficacy against different lifestyle of coagulase-negative Staphylococcus isolates from chronic bovine mastitis. Rep 8, 5081. doi: 10.1038/s41598-018-23521-0 PMC5865155 29572457

[B9] Cai Q. Wang G. B. Li Z. Zhang L. S. Fu Y. Y. Yang X. J.. (2019). SWATH based quantitative proteomics analysis reveals Hfq2 play an important role on pleiotropic physiological functions in Aeromonas hydrophila. J. Proteomics 195, 1–10. doi: 10.1016/j.jprot.2018.12.030 30597314

[B10] Colclough A. L. Scadden J. Blair J. M. A. (2019). TetR-family transcription factors in Gram-negative bacteria: conservation, variation and implications for efflux-mediated antimicrobial resistance. BMC Genomics 20, 731. doi: 10.1186/s12864-019-6075-5 31606035 PMC6790063

[B11] Cuthbertson L. Nodwell J. R. (2013). The TetR family of regulators. Microbiol. Mol. Biol. Rev. 77, 440–475. doi: 10.1128/MMBR.00018-13 24006471 PMC3811609

[B12] Damian S. Andrea F. Stefan W. Kristoffer F. Davide H. Jaime H. C.. (2015). STRING v10: protein–protein interaction networks, integrated over the tree of life. Nucleic Acids Res. 43, 447–452. doi: 10.1093/nar/gku1003 PMC4383874 25352553

[B13] Damian S. Gable A. L. David L. Alexander J. Stefan W. Jaime H. C.. (2018). STRING v11: protein-protein association networks with increased coverage, supporting functional discovery in genome-wide experimental datasets. Nucleic Acids Res. 47, D607–D613. doi: 10.1093/nar/gky1131 PMC6323986 30476243

[B14] D’Arpa P. Karna S. L. R. Chen T. Leung K. P. (2021). *Pseudomonas aeruginosa* transcriptome aDEPtations from colonization to biofilm infection of skin wounds. Sci. Rep. 11, 20632. doi: 10.1038/s41598-021-00073-4 34667187 PMC8526614

[B15] Dehbashi S. Tahmasebi H. Alikhani M. Y. Vidal J. E. Seifalian A. Arabestani M. R. (2023). The healing effect of Pseudomonas Quinolone Signal (PQS) with co-infection of Staphylococcus aureus and Pseudomonas aeruginosa: A preclinical animal co-infection model. J. Infect. Public Health 17, 329–338. doi: 10.1016/j.jiph.2023.12.016 38194764

[B16] Deng W. Li C. Xie J. (2013). The underling mechanism of bacterial TetR/AcrR family transcriptional repressors. Cell. Signaling 25, 1608–1613. doi: 10.1016/j.cellsig.2013.04.003 23602932

[B17] De Silva L. A. D. S. Wickramanayake M. V. K. S. Heo G. J. (2021). Virulence and antimicrobial resistance potential of Aeromonas spp. associated with shellfish. Lett. Appl. Microbiol. 73, 176–186. doi: 10.1111/lam.13489 33891720

[B18] Dias C. Borges A. Saavedra M. J. SimÕs M. (2017). Biofilm formation and multidrug resistant Aeromonas spp. from wild animals. J. Global Antimicrobial Resistance 12, 227–234. doi: 10.1016/j.jgar.2017.09.010 28951073

[B19] Dorick J. M. Macarisin D. Dunn L. Dev Kumar G. (2023). Effect of aquaponic water and substratum material on biofilm formation by *Aeromonas hydrophila* . Int. J. Food Microbiol. 404, 110316. doi: 10.1016/j.ijfoodmicro.2023.110316 37499272

[B20] Elbehiry A. Marzouk E. Abdeen E. Al-Dubaib M. Alsayeqh A. Ibrahem M.. (2019). Proteomic characterization and discrimination of Aeromonas species recovered from meat and water samples with a spotlight on the antimicrobial resistance of *Aeromonas hydrophila* . Microbiol. Open 8, e782. doi: 10.1002/mbo3.782 PMC6854848 30614207

[B21] Gallegos-Monterrosa R. Kankel S. Götze S. Barnett R. Stallforth P. Kovács Á. T. (2017). Lysinibacillus fusiformis M5 induces increased complexity in *Bacillus subtilis* 168 colony biofilms via hypoxanthine. J. Bacteriol. 199, e00204–e00217. doi: 10.1128/JB.00204-17 28583948 PMC5648860

[B22] Hall D. C. Król J. Cahill J. P. Ji H. F. Ehrlich G. D. (2020). The development of a pipeline for the identification and validation of small-molecule RelA inhibitors for use as anti-biofilm drugs. Microorganisms 8, 1310. doi: 10.3390/microorganisms8091310 32872142 PMC7563162

[B23] Kendall S. L. Burgess P. Balhana R. Withers M. Ten Bokum A. Lott J. S.. (2010). Cholesterol utilization in mycobacteria is controlled by two TetR-type transcriptional regulators: *kstR* and *kstR2* . Microbiology 156, 1362. doi: 10.1099/mic.0.034538-0 20167624 PMC3068626

[B24] Kim J. Park H. D. Chung S. (2012). Microfluidic approaches to bacterial biofilm formation. Molecules 17, 9818–9834. doi: 10.3390/molecules17089818 22895027 PMC6268732

[B25] Kumar J. Kumar M. Sharma S. Srivastava N. Singh R. Hussain M. A. (2022). Th1-Th2 and M1-M2 interplay sculpt Aeromonas hydrophila pathogenesis in zebrafish (*Danio rerio*). Fish Shellfish Immunol. 127, 357–365. doi: 10.1016/j.fsi.2022.06.052 35772676

[B26] Kussell E. Leibler S. (2005). Phenotypic diversity, population growth, and information in fluctuating environments. Science 309, 2075–2078. doi: 10.1126/science.1114383 16123265

[B27] Levin B. R. Rozen D. E. (2006). Non-inherited antibiotic resistance. Nat. Rev. Microbiol. 4, 556–562. doi: 10.1038/nrmicro1445 16778840

[B28] Li H. Li Y. Sun T. Du W. Ding C. (2020). Integrative proteome and acetylome analyses of murine responses to *Cryptococcus neoformans* infection. Front. Microbiol. 11, 575. doi: 10.3389/fmicb.2020.00575 32362878 PMC7181412

[B29] Li W. Yao Z. Sun L. Hu W. Cao J. Lin W.. (2016). Proteomics analysis reveals a potential antibiotic cocktail therapy strategy for *Aeromonas hydrophila* infection in biofilm. J. Proteome Res. 15, 1810–1820. doi: 10.1021/acs.jproteome.5b01127 27110028

[B30] Lin G. Chen W. Su Y. Qin Y. Huang L. Yan Q.. (2017). Ribose operon repressor (RbsR) contributes to the adhesion of *Aeromonas hydrophila* to *Anguilla japonicamucus* . Microbiol. Open 6, e00451. doi: 10.1002/mbo3.451 PMC5552941 28127946

[B31] Park A. J. Murphy K. Krieger J. R. Brewer D. Taylor P. Habash M.. (2014). A temporal examination of the planktonic and biofilm proteome of whole cell *Pseudomonas aeruginosa* PAO1 using quantitative mass spectrometry. Mol. Cell. Proteomics 13, 1095–1105. doi: 10.1074/mcp.M113.033985 24532839 PMC3977187

[B32] Paz L. Lemos J. A. Wickström C. Sedgley C. M. (2012). Role of (p)ppGpp in biofilm formation by *Enterococcus faecalis* . Appl. Environ. Microbiol. 78, 1627. doi: 10.1128/AEM.07036-11 22179256 PMC3294496

[B33] Pires R. H. Cataldi T. R. Franceschini L. M. Labate M. V. Fusco-Almeida A. M. Labate C. A.. (2016). Metabolic profiles of planktonic and biofilm cells of *Candida orthopsilosis* . Future Microbiol. 11, 1299–1313. doi: 10.2217/fmb-2016-0025 27662506

[B34] Pometun A. A. Boyko K. M. Yurchenko T. S. Nikolaeva A. Y. Kargov I. S. Atroshenko D. L.. (2020). Highly-active recombinant formate dehydrogenase from pathogenic bacterium *Staphylococcus aureus*: preparation and crystallization. Biochem. (Moscow) 85, 689–696. doi: 10.1134/S0006297920060061 32586232

[B35] Rasmussen-Ivey C. R. Figueras M. J. McGarey D. Liles M. R. (2016). Virulence factors of aeromonas hydrophila: in the wake of reclassification. Front. Microbiol. 7, 1337. doi: 10.3389/fmicb.2016.01337 27610107 PMC4997093

[B36] Rkenes T. P. Lamark T. Strøm A. R. (1996). DNA-binding properties of the BetI repressor protein of *Escherichia coli*: the inducer choline stimulates BetI-DNA complex formation. J. bacteriol. 178, 1663–1670. doi: 10.1128/jb.178.6.1663-1670.1996 8626295 PMC177852

[B37] Ruan X. Deng X. Tan M. Wang Y. Hu J. Sun Y.. (2021). Effect of resveratrol on the biofilm formation and physiological properties of avian pathogenic *Escherichia coli* . J. Proteomics 249, 104357. doi: 10.1016/j.jprot.2021.104357 34450330

[B38] Schujman G. E. Paoletti L. Grossman A. D. Mendoza D. D. (2003). FapR, a Bacterial transcription factor involved in global regulation of membrane lipid biosynthesis. Dev. Cell. 4, 663–672. doi: 10.1016/S1534-5807(03)00123-0 12737802

[B39] Seike S. Kobayashi H. Ueda M. Takahashi E. Yamanaka H. (2021). Outer membrane vesicles released from *Aeromonas* strains are involved in the biofilm formation. Front. Microbiol. 11, 613650. doi: 10.3389/fmicb.2020.613650 33488556 PMC7817658

[B40] Shannon P. Markiel A. Ozier O. Baliga N. S. Wang J. T. Ramage D.. (2003). Cytoscape: a software environment for integrated models of biomolecular interaction networks. Genome Res. 13, 2498–2504. doi: 10.1101/gr.1239303 14597658 PMC403769

[B41] Shu Y. C. Ho K. L. Cheah Y. K. Ng T. S. Than L. (2019). Glyoxylate cycle gene ICL1 is essential for the metabolic flexibility and virulence of *Candida glabrata* . Sci. Rep. 9, 2843. doi: 10.1038/s41598-019-39117-1 30808979 PMC6391369

[B42] Smitha S. Arpita G. Sanjay K. Johnson D. T. Anne G. (2021). Similar solutions to a common challenge: regulation of genes encoding *Ralstonia solanacearum* xanthine dehydrogenase. FEMS Microbiol. Letters 368, fnab022. doi: 10.1093/femsle/fnab022 PMC8324234 33620442

[B43] Solano C. Echeverz M. Lasa I. (2014). Biofilm dispersion and quorum sensing. Curr. Opin. Microbiol. 18, 96–104. doi: 10.1016/j.mib.2014.02.008 24657330

[B44] Vaysse P. J. Prat L. Mangenot S. Cruveiller S. Goulas P. Grimaud R. (2009). Proteomic analysis of *Marinobacter hydrocarbonoclasticus* SP17 biofilm formation at the alkane-water interface reveals novel proteins and cellular processes involved in hexadecane assimilation. Res. Microbiol. 160, 829–837. doi: 10.1016/j.resmic.2009.09.010 19786096

[B45] Venkatesan N. Perumal G. Doble M. (2015). Bacterial resistance in biofilm-associated bacteria. Future Microbiol. 10, 1743–1750. doi: 10.2217/fmb.15.69 26517598

[B46] Wang Y. Wang X. Ali F. Li Z. Fu Y. Yang X.. (2019). Comparative extracellular proteomics of *Aeromonas hydrophila* reveals iron-regulated secreted proteins as potential vaccine candidates. Front. Immunol. 10, 256. doi: 10.3389/fimmu.2019.00256 30833947 PMC6387970

[B47] Webber M. A. Ashraf T. Piddock L. (2018). Contribution of mutation at amino acid 45 of AcrR to acrB expression and ciprofloxacin resistance in clinical and veterinary Escherichia coli isolates. Antimicrob. Agents Chemother. 49, 4390–4392. doi: 10.1128/AAC.49.10.4390-4392.2005 PMC1251554 16189130

[B48] Wisniewski J. R. (2016). Quantitative evaluation of filter aided sample preparation (FASP) and multienzyme digestion FASP protocols. Analytical Chem. 88, 5438–5443. doi: 10.1021/acs.analchem.6b00859 27119963

[B49] Zhang L. Chen X. Wang G. Yao J. Wei J. Liu Z.. (2022). Quantitative proteomics reveals the antibiotics aDEPtation mechanism of Aeromonas hydrophila under kanamycin stress. J. Proteomics 264, 104621. doi: 10.1016/j.jprot.2022.104621 35618212

[B50] Zhang L. Li W. Sun L. Wang Y. Lin Y. Lin X. (2020). Quantitative proteomics reveals the molecular mechanism of *Aeromonas hydrophila* in enoxacin stress. J. Proteomics 211, 103561. doi: 10.1016/j.jprot.2019.103561 31676434

[B51] Zhang L. Wen B. Bao M. Cheng Y. Mahmood T. Yang W.. (2021). Andrographolide sulfonate is a promising treatment to combat methicillin-resistant *Staphylococcus aureus* and its biofilms. Front. Pharmacol. 12, 720685. doi: 10.3389/fphar.2021.720685 34603031 PMC8481920

[B52] Zhang B. Zhuang X. Guo L. McLean R. J. C. Chu W. (2019). Recombinant N-acyl homoserine lactone-Lactonase AiiAQSI-1 Attenuates Aeromonas hydrophila Virulence Factors, Biofilm Formation and Reduces Mortality in Crucian Carp. Mar. Drugs 17, 499. doi: 10.3390/md17090499 31461929 PMC6780897

