# Supplementary material for: Quantitative proteomics analysis reveals an important role of the transcriptional regulator UidR in the bacterial biofilm formation of Aeromonas hydrophila
Source: Front Cell Infect Microbiol. 2024 Mar 22;14:1380747. doi: 10.3389/fcimb.2024.1380747 (PMC10995333; doi:10.3389/fcimb.2024.1380747)

**Supplementary Figures and Tables**

**Supplementary Figure S1. The construction of** ***uidR* complemented mutant strain in *A. hydrophila*.** Lane 1: the fragments of target gene DNA amplified using the verification primers F and R in *uidR* complemented strain. M: DL2000 maker.

**Supplementary Figure S2. GO analysis of altered proteins between ΔuidR and WT strain in biofilm.** Enrichment analysis of differentially expressed proteins in the cell component.

**Figure S1**


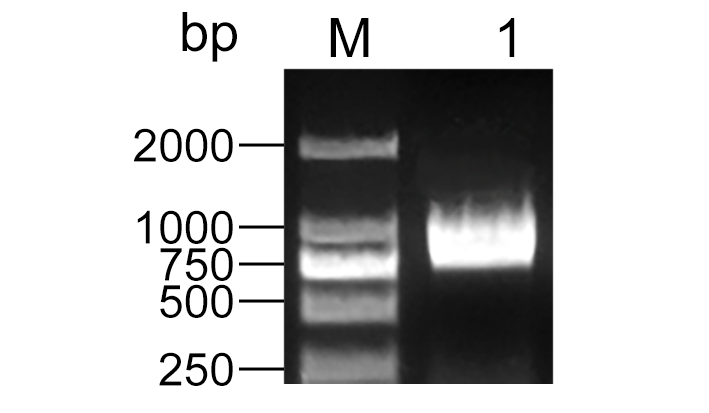


**Figure S2.**


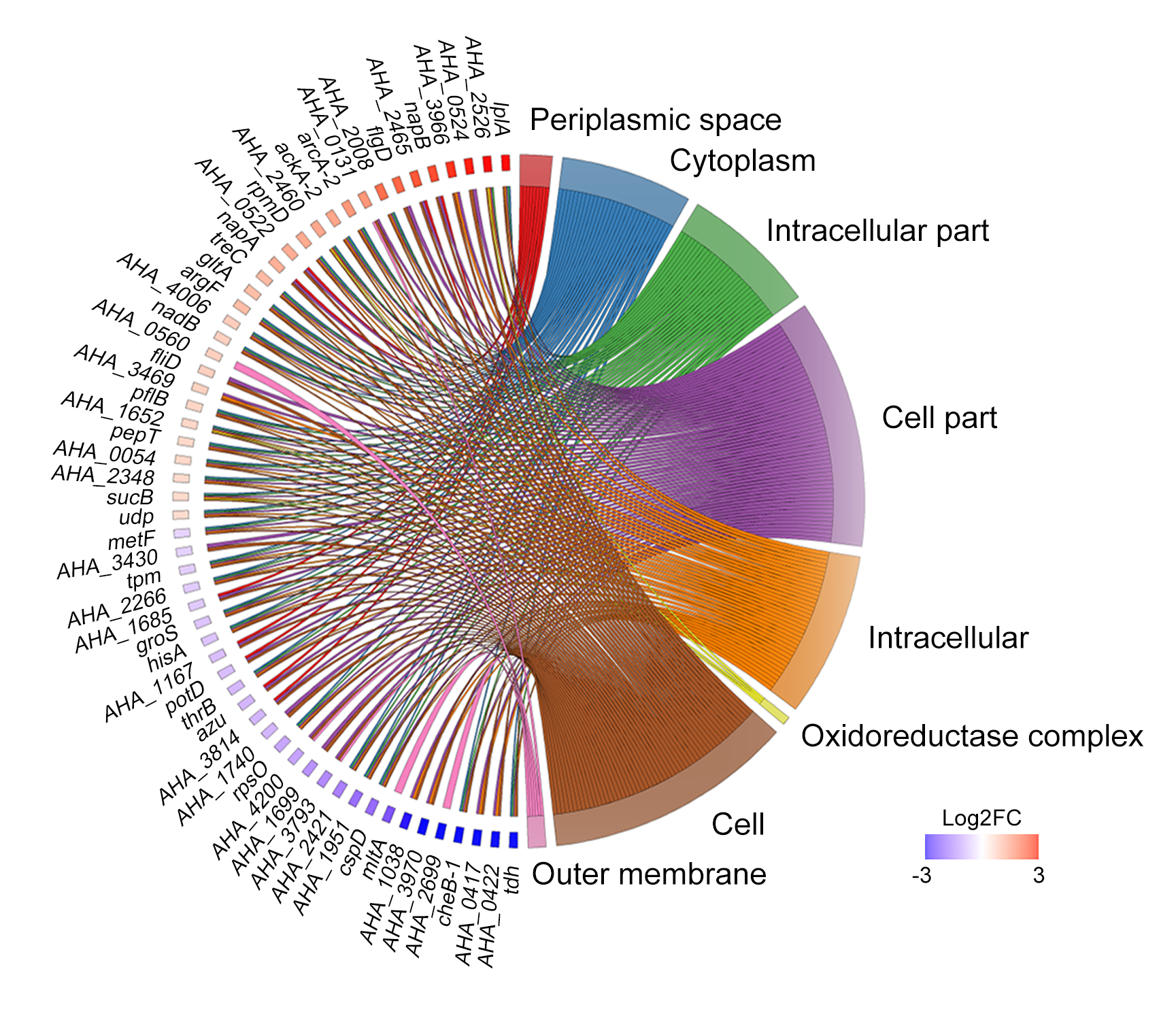

Supplement: Supplementary Table 1 — The strains and plasmids used in this study. [file DataSheet_1.docx]
